# The Role of Adenovirus in Cancer Therapy

**DOI:** 10.3390/cancers12113121

**Published:** 2020-10-26

**Authors:** Mizuho Sato-Dahlman, Brett Lee Roach, Masato Yamamoto

**Affiliations:** 1 Division of Basic and Translational Research, Department of Surgery, University of Minnesota, MMC 195, 420 Delaware St SE, Minneapolis, MN 55455, USA; satom@umn.edu (M.S.-D.); roach229@umn.edu (B.L.R.); 2 Masonic Cancer Center, University of Minnesota, Minneapolis, MN 55455, USA; 3 MCDB&G Program, College of Biological Sciences, University of Minnesota, Minneapolis, MN 55455, USA; 4 Institute of Molecular Virology, University of Minnesota, Minneapolis, MN 55455, USA

This series of 13 articles (7 original articles, 6 reviews) is presented by international leaders in adenovirus-based cancer therapy. Virotherapy strategies provide new options for treatment of various diseases, including cancer. Oncolytic adenovirus (OAd) is one of the most promising anti-cancer agents, and it has been employed for anti-tumoral potency via its strong oncolytic effect and intratumoral amplification. Additionally, virus-mediated cell lysis releases tumor antigens and induces local inflammation (e.g., immunogenic cell death), which contributes significantly to the reversal of local immune suppression and development of antitumor immune responses (“cold” tumor into “hot” tumor). Thus, OAd-based therapy is becoming increasingly popular for the treatment of many different forms of cancer. This Special Issue features recent advances in adenovirus vector technologies, as well as combinations with other therapies, toward clinical application.

Among many clinical trials with OAd-based therapies, Ad2 and Ad5 serotypes are most widely used and studied for oncolytic therapy. Although their biology is very well characterized and they can effectively transfer genes in vitro and in vivo, various limitations still exist. For instance, their infectivity is extremely low in many cancers poorly expressing the adenoviral primary receptor (Coxsackie adenovirus receptor, CAR). The efficacy is also potentially hampered by neutralizing antibodies, hampering the use of conventional adenovirus-based vectors for systemic injection. To address these issues, several of the articles discussed that various strategies have been employed and demonstrated to be effective in achieving a more specific targeting of cancer cells: for example, de-targeting and specific re-targeting modifications in the host-interacting domains of the adenovirus protein, such as HVR regions of hexon and AB-loop or HI-loop regions of fibers [1]. As a new approach of enhancing CAR-independent transduction of cancer cells, Ehrhardt’s group demonstrated the propensity of alternative adenovirus to transduce target cells via another receptor other than CAR [2]. They compared more than 20 Ad types representing species B1, B2, C, D, E, and G regarding their ability to transduce human breast cancer cell lines and breast epithelia cells. Ad3, Ad35, Ad37, and Ad52 were identified as potential candidates for breast cancer virotherapy, and these Ad types use alternative cellular receptors to achieve CAR-independent infection [3]. Moreover to enhancing the cancer-specific viral replication and killing, conditionally replicative adenoviruses (CRAds) have been developed. There are two main types of CRAds: mutation-based and cancer-specific promoter-based [1,2]. The first type of CRAds utilizes mutations or deletions in the E1 region of the adenoviral genome, which allows replication only in specific tumors which can compensate the loss of function due to mutation. ONYX-015 is an OAd that lacks the E1B region, designed to selectively replicate in mutated p53 tumors. Similarly, Ad∆24 is an OAd with a mutation in E1A and restricts replication to retinoblastoma protein (pRb) mutated cancer cells [1]. In this series, Tazawa et al. discuss the promoter-based CRAds. Specifically, they focus on the therapeutic potential of three types of telomerase (hTERT)-specific CRAds against bone and soft-tissue sarcoma cells with telomerase activity [4]. Extending the concept of viral replication-controlled CRAds, Higashino’s group demonstrated that inserted adenylate-uridylate-rich elements (AREs) from two human genes, a stabilizing element found in a type of macromolecule present in all biological cells, into OAd (AdARET and AdAREF) helps to specifically attack cancer cells [5]. Moreover, they showed that Paclitaxel treatment synergistically enhanced the oncolytic activity of AdAREF (also known as Ad-fosARE) both in vitro and in vivo [6], and also demonstrated that the combination of adenovirus and Cisplatin increased cell killing and increased virus replication both in vitro and in vivo [7]. In terms of combination therapy, another interesting report in this issue by Pokrovska et al. highlights the potential benefits of combining radiation therapy with oncolytic viruses [8]. In vivo experiments using xenograft models in SCID mice showed an additive, and possibly a synergistic, effect when combining EnAd (hybrid of two group B adenoviruses—Ad3 and Ad11p) with low-dosage radiation. This study complements previous findings of OAd having the potential to serve as radiosensitizers [8].

While many preclinical studies of OAd-based therapy are promising, clinical results of OAd monotherapies show limited efficacy, so far. There is a growing body of evidence from recent preclinical and clinical studies indicating that the host immune response may provide a critical boost for the efficacy of oncolytic virotherapy. Indeed, Franco-Luzón et al. reported Celyvir (ICOVIR-5 oncolytic adenovirus delivered by autologous mesenchymal stem cells) therapy caused increased infiltration and changes in the quality of immune cells per unit of tumor volume when compared to untreated mice. After this treatment, the tumor microenvironment showed a less protumoral and more inflammatory profile [9]. Yousaf et al. also suggest that adenovirus infection could contribute to a localized reduction in vascular perfusion, and perhaps even to the alleviation of immunosuppression [10]. Sato-Dahlman et al. discuss recent progress in the combination therapy of adenovirus with an immunotherapeutic reagent, such as immune checkpoint inhibitors and CAR-T cells, in preclinical and clinical studies [11]. Additionally, McKenna et al. also discuss how more accurate in vitro and in vivo models can evaluate the ability of OAds to not only induce oncolysis but also penetrate the solid tumor extracellular matrix and stimulate an immune response [12].

The collection also includes insightful reviews discussing the important features needed to be considered when expressing transgenes from oncolytic adenoviruses, also known as “arming OAds” [13]. The insertion of therapeutic transgenes into OAds genomes has been the main strategy to improve their therapeutic potential. However, one must consider at least four different parameters when designing an armed OAd: transgene location, transcriptional control elements, transgene codon usage, and the transgene itself. For example, they postulated that the E3 insertion site could be beneficial for immunocentric transgenes aiming to stimulate the immune system due to the elimination of E3’s inherent functions that are specifically involved in immune evasion [13]. On the other hand, when expressing virocentric transgenes, which aim to improve the oncolytic effect, they suggest expressing these in late transcription sites (L1–4) in non-E3-deleted viruses for a longer persistency [13]. Overall, a strategy arming OAds must consider numerous factors including these major factors in order to yield desired outcomes. 

From the 13 high-quality papers and reviews, we observe the diversity of approaches and great potential of oncolytic adenovirus as a cancer therapy agent. The flexibility of DNA manipulation to drive de-targeting and re-targeting of adenoviruses, combined with conditional replication and targeted expression, allows for the combination of multiple useful tools into one. In order to enhance the therapeutic effect of OAds and improve the patient outcome, there is a shift in the OAd therapy field away from OAd monotherapy and toward combining OAd with additional synergistic strategies: radiotherapy, chemotherapies, immune checkpoint blockade, or the use of other cellular therapies including CAR-T cells. We hope that this Special Issue will provide new and significant inspiration for future research of adenovirus-based cancer therapy.
